# Total eclipse in the heart

**DOI:** 10.1007/s12471-017-1059-z

**Published:** 2017-12-14

**Authors:** R. J. de Winter

**Affiliations:** Department of Cardiology, Academic Medical Center, Amsterdam, The Netherlands

## Answer

### Aorto-right atrial tunnel (ARAT)

The entrance of the tunnel arises from one of the coronary cusps of the aorta. Because this structure may arise from the non-coronary cusp, this is not considered to be a coronary fistula [1]. The tunnel connects with the right atrium. Closing the ARAT with vascular plugs is achieved as follows: The entrance of the tunnel is visualised from the aorta. A wire is passed down and snared in the right atrium or superior caval vein and a body loop is formed. Careful positioning of a delivery sheath from the venous side into the tunnel is performed, without traction on the entrance of the tunnel or the aorta. Large size vascular plugs are often needed.
